# Obese Adipose Tissue as a Driver of Breast Cancer Growth and Development: Update and Emerging Evidence

**DOI:** 10.3389/fonc.2021.638918

**Published:** 2021-03-30

**Authors:** Priya Bhardwaj, Kristy A. Brown

**Affiliations:** ^1^ Department of Medicine, Weill Cornell Medicine, New York, NY, United States; ^2^ Graduate School of Medical Sciences, Weill Cornell Medicine, New York, NY, United States; ^3^ Meyer Cancer Center, Weill Cornell Medicine, New York, NY, United States

**Keywords:** obesity, breast cancer, development, growth, estrogen

## Abstract

Obesity is an established risk factor for breast cancer growth and progression. A number of advances have been made in recent years revealing new insights into this link. Early events in breast cancer development involve the neoplastic transformation of breast epithelial cells to cancer cells. In obesity, breast adipose tissue undergoes significant hormonal and inflammatory changes that create a mitogenic microenvironment. Many factors that are produced in obesity have also been shown to promote tumorigenesis. Given that breast epithelial cells are surrounded by adipose tissue, the crosstalk between the adipose compartment and breast epithelial cells is hypothesized to be a significant player in the initiation and progression of breast cancer in individuals with excess adiposity. The present review examines this crosstalk with a focus on obese breast adipose-derived estrogen, inflammatory mediators and adipokines, and how they are mechanistically linked to breast cancer risk and growth through stimulation of oxidative stress, DNA damage, and pro-oncogenic transcriptional programs. Pharmacological and lifestyle strategies targeting these factors and their downstream effects are evaluated for feasibility and efficacy in decreasing the risk of obesity-induced breast epithelial cell transformation and consequently, breast cancer development.

## Introduction

Breast cancer is the second most common cancer diagnosed in women in the U.S. and the most common cancer among women worldwide, accounting for an estimated 627,000 deaths in 2018 (1, 2). In 2020, over a quarter million breast cancers were diagnosed in the U.S., representing a slow but steady increase in new diagnoses over the last 30 years (3). There are a number of well-established risk factors that impact the prevalence of breast cancer, including genetics, age, reproductive history, breast density, and exposure to hormones (4, 5). Additionally, lifestyle factors like alcohol consumption, physical inactivity, and elevated bodyweight have been causally linked with breast cancer development and worse prognosis (6, 7). The link between elevated bodyweight and breast cancer is of particular significance in light of the rapidly rising rates of obesity worldwide, nearly tripling since 1975 (8). By 2025 global obesity prevalence is expected to reach 18% in men and surpass 21% in women (8), with some estimates predicting significantly faster growth (9). Meanwhile, the U.S. has already seen obesity rates reaching 42.4% as of 2018, with the highest rates observed in women (10). Therefore, understanding the mechanistic basis of how obesity contributes to elevated risk of breast cancer and worse outcomes are critical from the standpoint of prevention and management.

## The Relationship Between Obesity and Breast Cancer

Obesity, as defined by a body mass index (BMI, kg/m^2^) greater than or equal to 30, has consistently been associated with breast cancer in postmenopausal women. In premenopausal women, an inverse association has been observed and attributed in part to higher rates of amenorrhea in obese women and consequently, decreased circulating estrogen levels (11, 12). However, among postmenopausal women, there is a strong causal relationship between obesity and estrogen receptor positive (ER+) breast cancer (13, 14). In 2016, the International Agency for Research on Cancer (IARC) conducted a systematic review of over 1000 epidemiological studies on the risk of cancer due to excess body fat. This large-scale review confirmed the relationship between obesity and postmenopausal breast cancer while also concluding that there was substantial evidence establishing an association between increased BMI and reduced survival among breast cancer patients (15). The composition of the breast lends some insight into how obesity may be mechanistically linked with breast cancer. The breast is comprised of three major components: fibrous tissue, glandular tissue, and fat (adipose) tissue. On a cellular level, the adipose tissue compartment consists of adipocytes that store lipid, preadipocytes (adipose stromal cells), immune cells, and endothelial cells. The glandular tissue refers to the epithelial cells that form lobules and ducts, which produce and transport milk within the breast, respectively. The cells within these compartments are plastic as the breast undergoes significant changes during development, pregnancy, and in response to environmental factors. For example, during cold exposure white adipocytes can undergo “browning” and differentiate into beige adipocytes which have thermogenic properties when activated (16). Additionally, during pregnancy secretory alveolar mammary epithelial cells expand to support lactation and undergo involution *via* apoptosis during the post-lactation period (17). Studies in pregnant mice have suggested the ability of white adipocytes to transdifferentiate into milk producing mammary epithelial cells containing lipid droplets and termed “pink” adipocytes (18).

**Figure 1:**
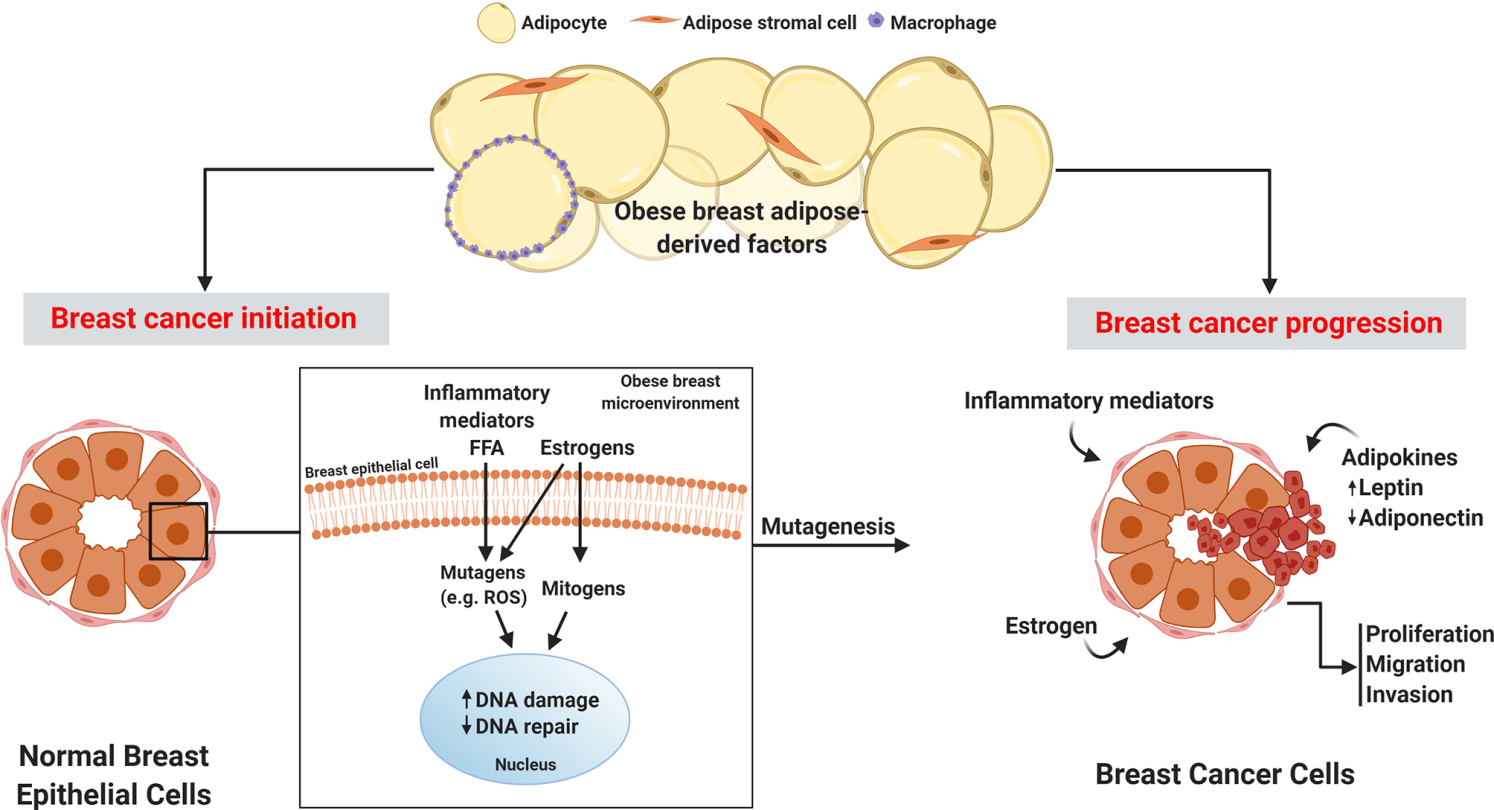
Obese breast adipose-derived factors contribute to the initiation and progression of breast cancer. Factors produced by obese breast adipose tissue including inflammatory mediators, free fatty acids (FFA), and estrogens can act as mutagens, for example by stimulating intracellular reactive oxygen species (ROS), which can lead to DNA damage in normal breast epithelial cells. Estrogen also has mitogenic effects that can lead to replication stress and consequently DNA damage. Elevation in DNA damage coupled with potential estrogen-induced dysfunctional DNA repair may lead to unresolved DNA damage, which is linked to mutagenesis and initiation of cancer. Breast cancer progression is fueled by inflammatory mediators, excess leptin, decreased adiponectin, and elevated estrogens in the obese breast adipose microenvironment which promote proliferation, migration, and invasion of breast cancer.

During breast carcinogenesis, it is the epithelial cells that undergo neoplastic transformation into cancer cells. Notably, breast epithelial cells are embedded in adipose tissue, allowing for paracrine interaction between epithelial cells and cells within the adipose compartment. In a recent study, microdissection was performed on epithelium, adipose tissue, and stroma in breast sections from women before and after breast cancer diagnosis. Co-expression network analyses comparing tissue associations between compartments showed increased interaction between the epithelial compartment and surrounding adipose tissue prior to breast cancer development, confirming the existence of a crosstalk between breast epithelial cells and adjacent adipose tissue (19). This is a significant finding since in the setting of obesity, breast adipose tissue undergoes considerable dysregulation in the production of estrogens, adipokines, inflammatory mediators, and reactive oxygen species (ROS), creating a microenvironment primed for initiating breast cancer development and promoting progression **(** **Figure 1** **)**.

## Obesity-Induced Dysregulation of the Breast Adipose Microenvironment

### Estrogens

Estrogens, a class of steroid hormones, are major drivers of obesity-associated ER+ breast cancer. While they play an important role in normal mammary gland development and in metabolic processes, excessive or dysregulated estrogen signaling drives ER+ breast cancer through both genomic and non-genomic actions (20, 21). 17β-estradiol (E2), the most potent endogenously produced estrogen, binds to its receptor, ERα, causing dimerization, translocation to the nucleus and binding to estrogen response elements (EREs) on target genes to drive transcription of a variety of programs that promote cancer growth, including cell proliferation and decreased apoptosis (4, 22, 23). Estrogens can also have receptor-independent mutagenic effects, as reviewed in the next section. Lifetime exposure to estrogens, and especially high levels after menopause, has been consistently linked with elevated breast cancer risk (4, 24).

**Figure 2:**
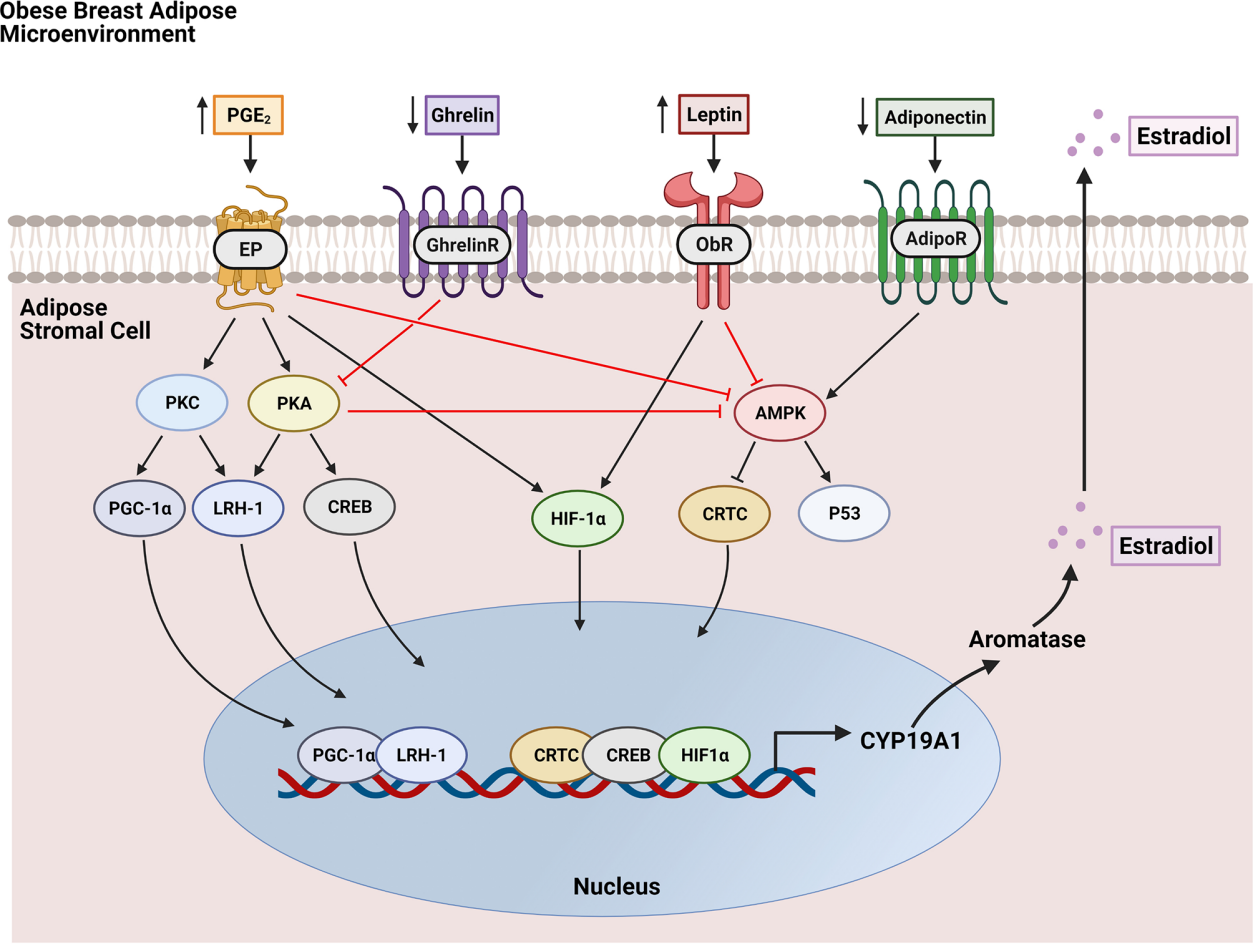
Regulation of aromatase expression in breast adipose stromal cells by the obese breast microenvironment. Hallmarks of the obese breast microenvironment include elevated PGE_2_ and leptin along with decreases in ghrelin and adiponectin. PGE_2_ binds EP receptors to activate signaling through protein kinase C (PKC) and protein kinase A (PKA) which results in increased expression and nuclear localization of transcription factor co-activator PGC-1α and transcription factors LRH-1 and CREB which both drive aromatase (CYP19A1) transcription. PGE_2_ also enhances aromatase expression through stimulation of HIF-1α-driven aromatase transcription and through inhibition of AMPK. AMPK activation negatively regulates aromatase by inhibiting entry into the nucleus of the aromatase transcription factor CRTC and by stimulating P53, which has been shown to translocate to the nucleus and inhibit aromatase transcription. Leptin signaling through its receptor ObR also increases HIF-1α expression and nuclear localization while inhibiting AMPK signaling. Both ghrelin and adiponectin negatively regulate aromatase expression through inhibition of PKA and through activation of AMPK, respectively. However, these factors are downregulated in the obese breast microenvironment, thereby decreasing their inhibitory effects on aromatase expression. The net effect of increased aromatase expression catalyzes the production of estradiol, which is released into the breast microenvironment.

Before menopause, E2 is produced predominantly by the ovaries and is released into circulation. After menopause, ovarian estrogen production ceases and circulating levels diminish. However, it is still synthesized to a lesser extent in peripheral tissues including bone, brain, vascular tissue, and mainly, adipose tissue (25). In obesity, adipose tissue levels of E2 are elevated due to increased adipose stromal cell (ASC) expression of the enzyme aromatase, which catalyzes the conversion of androstenedione to estrone (E1), and testosterone to E2 (26–28). In addition to aromatase expression increasing in ASCs from obese individuals, it has also been suggested that the number of ASCs within the breast increases as adipose tissue expands in obesity, further compounding the elevation in aromatase levels and consequently, estrogen levels in postmenopausal breast tissue (29–32). Aromatase expression in ASCs is modulated by the adipose tissue microenvironment, including the presence of tumors (22) **(** **Figure 2** **).** For example, both tumors and macrophages in obese breast adipose tissue secrete the inflammatory mediator prostaglandin E_2_ (PGE_2_) which was shown to increase expression and nuclear localization of hypoxia inducible factor-1α (HIF-1α) which in turn, stimulates promoter II-driven aromatase expression in ASCs (33). PGE_2_ also stimulates aromatase expression through inhibition of 5’ AMP-activated protein kinase (AMPK), a negative regulator of aromatase, and by increasing expression and nuclear localization of transcription factors cAMP response element-binding protein (CREB), liver receptor homolog-1 (LRH-1), and transcription factor co-activator peroxisome proliferator-activated receptor gamma coactivator 1-alpha (PGC-1α) which drive aromatase expression (23). The effects of PGE_2_ on aromatase expression in ASCs is further enhanced in the setting of obesity due to decreased expression of the gut-derived hormone ghrelin, which inhibits PGE_2_ signaling (34). Furthermore, obesity-induced modulation in the adipokines leptin and adiponectin results in a net inhibition of AMPK (35). Collectively, this increase in ASC aromatase expression leads to elevated estradiol production, which can fuel growth of nearby ER+ breast tumors. A recent study highlighted the importance of ASC-breast tumor crosstalk using an *ex vivo* organotypic breast model where mammary ASCs from lean and obese women were isolated and co-cultured with ER+ MCF7 breast cancer cells. It was demonstrated that ERE transactivation was heightened in MCF7 cells co-cultured with obese ASCs compared with lean ASCs due to increased expression of aromatase in obese ASCs (31).

### Inflammatory Mediators

**Figure 3:**
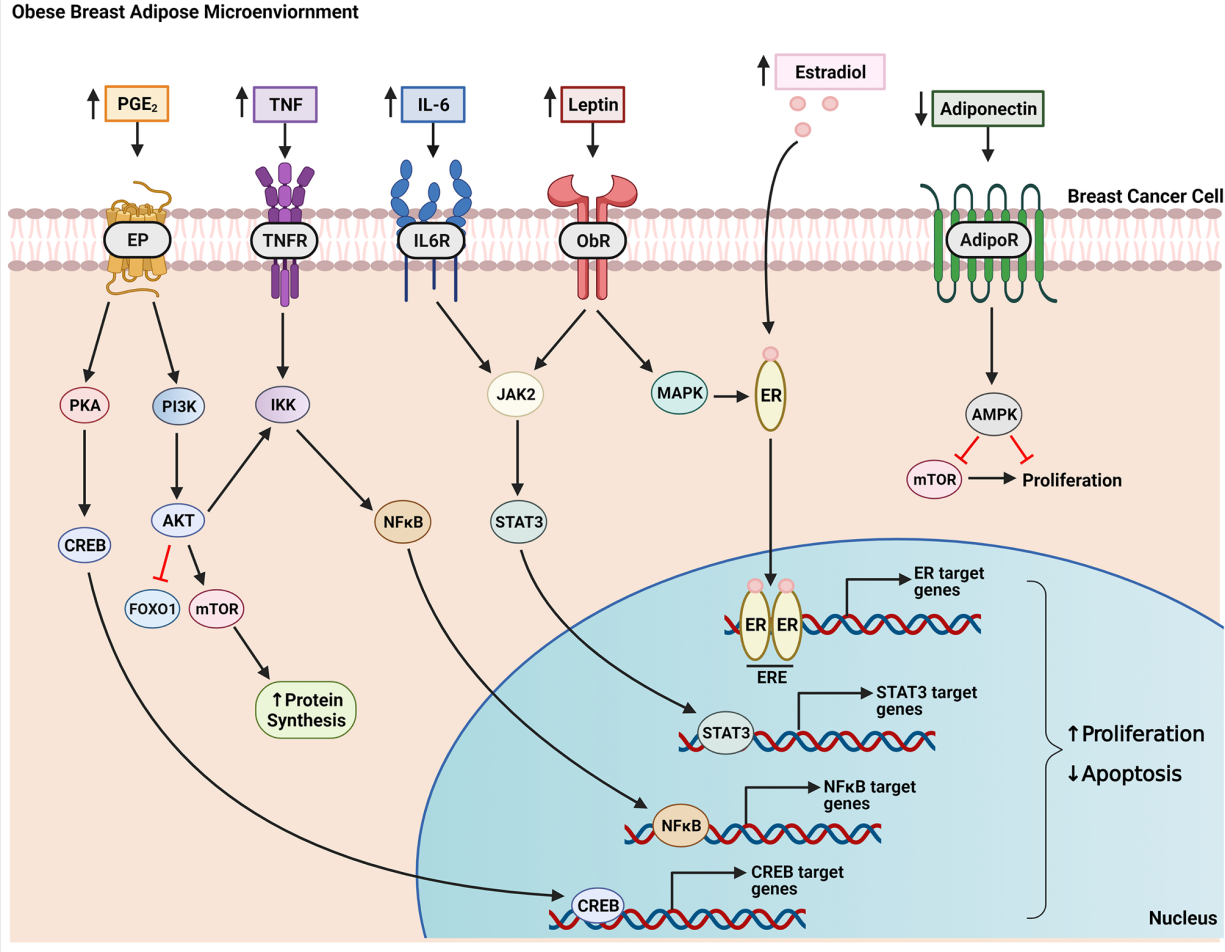
Factors produced in the obese breast microenvironment contribute to the growth of breast cancer. Obesity-induced changes to breast adipose tissue results in increased secretion of inflammatory mediators (e.g. PGE_2_, TNF, IL-6) and estradiol along with altered adipokine production. These factors act in a paracrine manner on breast tumor cells growing within this milieu to increase proliferation and decrease apoptosis. For example, PGE_2_ signals through EP receptors on tumor cells to activate PKA, which, in turn, stimulates CREB translocation to the nucleus and transcription of genes that promote cell growth. PGE_2_ also activates the PI3K pathway resulting in inhibition of FOXO1 and activation of mTOR *via* AKT, netting a decrease in apoptosis and increase in protein synthesis, a necessary precursor of cell growth. Both AKT and TNF signaling activate IKK leading to expression and translocation of transcription factor NFκB to the nucleus. IL-6 and leptin bind their respective receptors to activate JAK2/STAT3 signaling which promotes nuclear transcription of programs that enhance cell proliferation. Leptin also activates MAPK signaling which can lead to ligand-independent activation of the estrogen receptor (ER). Ligand-dependent (through binding of estradiol) or ligand-independent activation of ER causes receptor dimerization and binding to estrogen response elements (ERE) on target genes. Adiponectin signals through its receptor to activate AMPK signaling resulting in downstream inhibition of mTOR, which decreases proliferation. This inhibitory effect on cell proliferation is diminished in obesity due to decreased adiponectin production by obese breast adipose tissue.

Adipose tissue expansion during weight gain and in obesity leads to enlarged, hypertrophic adipocytes as they fill with excess dietary lipids. This results in a hypoxic breast microenvironment as growing cells have higher oxygen demands and vascularization cannot keep up with the pace of adipose expansion (36, 37). Inadequate oxygen supply leads to adipocyte cell death which signals the infiltration macrophages to surround and engulf necrotic adipocytes, forming characteristic inflammatory foci termed “crown-like structures” (CLS) in white adipose tissue (38, 39). In a study of 107 breast cancer patients, there was a 3.2 and 6.9 times higher likelihood of CLS occurring in breast tissue from overweight and obese women, respectively, compared with healthy weight patients (40). Macrophages in CLS produce inflammatory mediators, including TNF, IL-1β, IL-6, and PGE_2_, which contributes to the characterization of obesity as a state of chronic low-grade inflammation (41–44). In addition to macrophages, adipocytes have also been shown to secrete inflammatory mediators (45). In an *in vitro* study, co-culture of adipocytes with breast cancer cells resulted in synergistically increased production of IL-6, IL-8, CXCL10, CCL2, and CCL5 in the culture media (46). This contributes to a microenvironment conducive to breast cancer growth as inflammatory cytokines have an established role in activating signaling pathways related to proliferation, invasion, and migration of breast cancer cells (47–51)**(** **Figure 3** **)**.

Saturated fatty acids released by hypertrophic adipocytes undergoing lipolysis can also activate inflammatory cytokine production *via* ligand-mediated activation of toll-like receptors 2 and 4 (TLR2, TLR4) in both adipocytes and macrophages (52–54). In murine studies, high fat diet fed TLR2 and TLR4 deficient mice are protected from adipose inflammation, reinforcing the importance of these receptors as mediators of obesity-induced adipose inflammation (55–57). Recent work by Nishimoto et al. presented a novel mechanism for the development of adipose inflammation in obesity. Using *in vitro* and *in vivo* models, they showed that TLR9 activation facilitates the interaction between macrophages and adipocytes by increasing macrophage expression of monocyte chemoattractant protein-1 (MCP-1), which is a chemokine that signals macrophages to dead adipocytes (58). Furthermore, they found that obesity-related dying adipocytes shed cell-free DNA (cfDNA) that serve as an endogenous ligand for TLR9 in macrophages, thereby establishing an adipocyte cell death→cfDNA→TLR9 axis that leads to macrophage accumulation in obese adipose tissue. These studies were conducted exclusively in visceral fat, while breast tissue is considered subcutaneous fat. Therefore, further studies are warranted to determine whether this axis exists across fat depots. If so, it would represent an intriguing target for reducing breast adipose inflammation in obese postmenopausal women to protect against breast cancer development, or in a therapeutic setting to improve prognosis after diagnosis.

### Adipokines

Beyond its role as a storage site for lipids, adipose tissue is recognized as an endocrine organ that secretes hormones called adipokines. Two major adipokines secreted by adipose tissue are leptin and adiponectin, which have widely been shown to promote or inhibit breast cancer, respectively, by directly interacting with breast cancer cells and by regulation of metabolic homeostasis in obesity.

Leptin is produced primarily by adipocytes and is secreted in abundance by obese adipose tissue as a function of adipocyte size, with levels correlating positively with BMI (59). While initially discovered in 1994 as a satiety hormone, it has since been shown to play a variety of roles in metabolic homeostasis, as an inflammatory mediator, and as a mitogen (60–62). Accordingly, elevated leptin levels are positively associated with breast cancer risk (63–65). Leptin acts by binding to its receptor, Ob-R, leading to signal transduction through phosphorylation of JAK2 and downstream activation of STAT3, MAPK/ERK1/2, AMPK and PI3K/AKT pathways. Activation of these pathways results in transcription of genes involved in cell growth, proliferation, angiogenesis and cell survival (62, 66–69) **(** **Figure 3** **)**. *In vitro* work by Saxena et al. showed in a panel of breast cancer cell lines that leptin promotes proliferation through phosphorylation of JAK/STAT3 leading to translocation of phospho-STAT3 to the nucleus and transcription of cyclin D1, an important mediator of cell cycle (70). In addition to promoting breast cancer growth through stimulation of proliferation, leptin may also inhibit apoptosis of breast cancer cells by downregulating pro-apoptotic genes (71). However, this effect is not consistent across breast cancer cell lines.

Additionally, leptin promotes ER+ breast cancer cell proliferation through modulation of estrogen levels and signaling. For example, leptin was shown to transactivate ERα *via* signaling through ERK1/ERK2 in MCF7 breast cancer cells (72) and increase estrogen production in the local microenvironment by stimulating aromatase expression in ASCs (73–75) **(** **Figure 2** **)**. Interestingly, another study also in MCF7 cells found that estrogen increases leptin and Ob-R mRNA (76), potentially representing a positive feedback loop between leptin and estrogen that amplifies expression of both in obesity. Strong et al. reported that leptin is not only secreted by adipocytes, but also by ASCs (77). Further, they showed that ASCs isolated from obese women had higher leptin expression than ASCs isolated from lean women. Co-culture of obese ASCs with several ER+ breast cancer cell lines increased proliferation, migration, and invasion, an effect that was lost after silencing leptin in the ASCs (77, 78).

Several studies have suggested a role for leptin in the early events that lead to cancer development. Using the non-neoplastic mammary epithelial cell line HMT-3522, Tenvooren et al. demonstrated that leptin disrupts apical-basal polarity of 3D cultured mammary acini *via* activation of PI3K/AKT signaling, an early hallmark of cellular transformation in breast cancer (79). Another study also implicated leptin in the early stages of breast cancer, showing that leptin treatment promotes epithelial-mesenchymal transition through activation of the kinases Src and FAK in MCF10A cells, a non-cancerous mammary epithelial cell line, which led to enhanced invasion through Matrigel (80). In a study of normal breast epithelium from women before and after breast cancer diagnosis compared with women who never developed breast cancer, leptin was upregulated 3.8 fold in the breast epithelium before cancer diagnosis, suggesting that increased leptin may be an early event associated with epithelial cell transformation (19).

Like leptin, adiponectin is an adipokine produced and secreted by adipocytes. However, unlike leptin, adiponectin is elevated in lean adipose tissue and decreases with obesity (23, 81). While leptin has pro-carcinogenic effects, adiponectin is inversely linked to breast cancer risk and aggressiveness (62, 82, 83). Adiponectin signals through ligand binding with its receptors AdipoR1 and AdipoR2 and recruitment of intracellular binding partner APPL1 which leads to downstream activation of the AMPK pathway and to a lesser extent p38 MAPK and PPARα pathways. It is both directly and indirectly protective against obesity-related breast cancer. Direct anti-cancer effects of adiponectin have been illustrated in several breast cancer cell lines where adiponectin inhibited proliferation, increased apoptotic response, and in one study, induced autophagic cell death (84–86). Indirect effects involve regulation of anti-inflammatory processes through stimulation of anti-inflammatory cytokine IL-10 and inhibition of inflammatory cytokine production from macrophages (87, 88). Additionally, adiponectin plays an important role as an insulin sensitizer through AMPK-mediated elevation in glucose utilization and increased fatty acid oxidation (89, 90). Therefore, obesity-induced elevation in breast adipose leptin and reduction in adiponectin play a significant role in establishing a breast microenvironment conducive to cellular transformation and tumor growth.

## Obese Breast Adipose-Derived Sources of DNA Damage

In addition to fueling the growth of breast cancer, obesity has also been hypothesized to drive breast cancer initiation **(** **Figure 1** **)**. A growing number of studies have highlighted an apparent genomic instability associated with obesity (91–94). This is significant since genomic instability can lead to mutations that lead to tumorigenesis (95). Genomic instability in the form of DNA damage has been demonstrated in various contexts in association with obesity in both preclinical and clinical studies. For example, in one study DNA damage in peripheral blood lymphocytes was measured utilizing the comet assay that quantitates breaks in DNA. Both BMI and waist circumference were positively associated with DNA damage (94). Similar findings were made in an aging study, where obesity was a stronger predictor of skeletal muscle DNA damage compared to aging (96). In preclinical work, obese zucker rats were found to have increased levels of DNA damage in several organ sites as measured by frequency of cells staining positive for phosphorylated histone variant H2AX (γH2AX), a marker of double strand breaks in DNA (97). While the mechanisms of obesity-driven DNA damage are incompletely understood, potential sources of DNA damage in obesity-related breast cancer include the elevation in breast adipose oxidative stress, which is a known inducer of DNA damage (98–100), and genotoxic effects of estrogen, produced in abundance in obese breast adipose tissue, as described in earlier sections.

### Oxidative Stress

Reactive oxygen species (ROS) is a byproduct of cellular metabolism with both endogenous and exogenous sources. A major intracellular source of ROS is the mitochondria as a result of increased oxidative phosphorylation (101, 102). Mitochondrial ROS production occurs by the leakage of electrons from complexes I and III of the electron transport chain (ETC) during respiration, resulting in generation of the ROS species superoxide, a precursor for other ROS species, including hydrogen peroxide and hydroxyl. Other endogenous sources of ROS include the NOX family of NADPH oxidases and nitric acid synthases. If left uncontrolled and out of balance with antioxidants, excessive ROS production leads to oxidative stress and DNA damage which has been extensively linked to the initiation of cancer (103–105).

A number of factors secreted by obese breast adipose tissue have the ability to stimulate ROS production. Inflammatory cytokines increase ROS production in both cancer and non-cancerous cells (48, 106, 107). For example, Kastl et al. found that stimulation of murine hepatocytes with TNF-α resulted in up to a 150% increase in ROS production from mitochondria complexes I and III without causing cell death (108). TNF-α administration can also increase intracellular ROS by depleting cytosolic and mitochondrial antioxidants (109). In chondrocytes, Ansari et al. showed that stimulation with IL-1β leads to mitochondrial dysfunction and elevated levels of ROS (110). IL-6 stimulates intracellular ROS production by decreasing membrane potential leading to more electron leakage from the electron transport chain (ETC) (111).

Additionally, adipocytes store fatty acids as triacylglycerol, which is hydrolyzed and released as free fatty acids (FFA) when needed as source of fuel, such as during fasting. In obesity, FFA levels are often elevated due to enhanced lipolysis of expanding adipocytes and reduced clearance. Cells utilize fatty acids as a fuel source *via* the process of β-oxidation in which fatty acids are broken down to produce energy (ATP). Fatty acid β-oxidation produces substrates that are used by the tricarboxylic acid (TCA) cycle and the mitochondrial ETC resulting in ATP production. Importantly, this process results in the release of ROS. Increased mitochondrial ROS production in several disease contexts has been demonstrated to be due to oxidation of fatty acids (112, 113). Interestingly, Yamagishi et al. demonstrated that leptin also induces mitochondrial ROS production by increasing fatty acid oxidation in endothelial cells (114). Since obesity is characterized by an increase in both leptin and FFAs, β-oxidation-induced mitochondrial ROS production may be enhanced even further with excess bodyweight. The ability of leptin to drive ROS formation has been validated in other studies as well. Non-cancerous primary human mammary epithelial cells (HMEC) upregulated ROS production when stimulated with leptin in a NOX5 dependent manner, even at relatively low doses (115). One study also proposed that leptin-mediated elevation in oxidative stress is due to reduction in the activity the antioxidant enzyme PON1 (116).

Abundant evidence demonstrates the ability of obese breast adipose-derived factors, including inflammatory mediators, fatty acids, and leptin, to elevate ROS and consequently oxidative stress. While it is conceivable that this elevation in breast microenvironment oxidative stress leads to DNA damage in pre-neoplastic breast epithelial cells, studies directly demonstrating this link are lacking. Further studies are warranted to determine whether an increase in ROS in obese breast adipose tissue represents a direct mechanistic link between obesity and breast cancer initiation.

### Estrogens

Estrogens stimulate DNA damage in three ways: 1) ligand binding to ERα stimulates proliferation which can lead to replication stress and accumulation of errors when the volume of replication errors overwhelms DNA damage repair capacity. Stimulation of proliferation also increases cellular respiration, which produces ROS as a byproduct of oxidative phosphorylation (4, 117, 118). 2) The metabolism of estrogen involves the redox cycling of catechol estrogen metabolites between semi-quinone and quinone states, a process that produces ROS (119–121). 3) Catechol estrogen metabolites can also directly interact with DNA to form mutagenic depurinating adducts, a form of DNA damage (122). For example, *in vitro* studies have demonstrated that the estrogen catechol metabolites 4-hydroxy-estradiol (4-OH-E2), 2-hydroxy-estradiol (2-OH-E2), and 2-methoxy-estradiol (2-MeO-E2) increase ROS and induce DNA damage in normal human mammary epithelial cells (123–125). Given the multiple avenues of estrogen-induced DNA damage, proficient DNA repair is crucial to limiting the propagation of damaged DNA. However, in addition to stimulating DNA damage, estrogens have also been shown to disrupt DNA repair machinery and consequently, response to damage (126, 127), further compounding the risk of DNA damage-induced mutagenesis due to excessive estrogen exposure, as in the setting of obesity.

## Prevention or Reversal of Obesity-Induced Adipose Dysfunction and DNA Damage as a Modifier of Breast Cancer Risk

**Figure 4:**
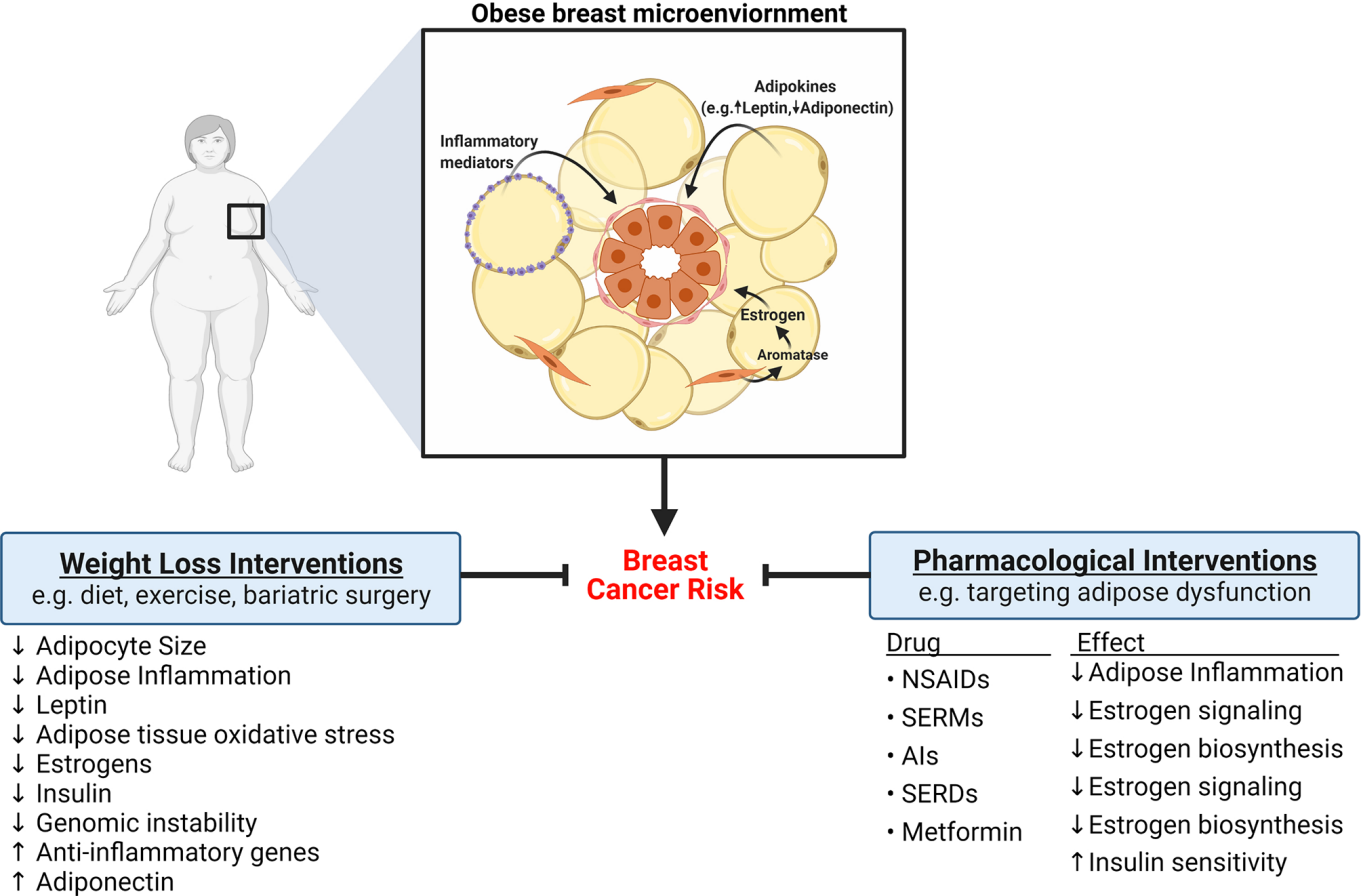
Strategies to reduce the risk of breast cancer development in obese women. Obesity is associated with significant changes to breast adipose tissue that increase the risk of developing breast cancer, including elevation in adipose inflammation, altered adipokine secretion, and elevation in estrogens. Ample evidence indicates that weight loss, achieved through diet, exercise, or bariatric surgery, can reverse or diminish some obesity-induced changes to adipose tissue and consequently reduce the risk of developing breast cancer. Pharmacological interventions that target adipose dysfunction have also been shown to be efficacious at reducing breast cancer risk. For example, nonsteroidal anti-inflammatory drugs (NSAIDs) decrease adipose inflammation. Drugs like selective estrogen receptor modulators (SERMS), aromatase inhibitors (AIs), and selective estrogen receptor degraders (SERDS), exert protective effects by inhibiting estrogen signaling. Metformin may also reduce risk of breast cancer through regulation of estrogen biosynthesis and improvement of insulin sensitivity.

Targeting obesity-induced changes in breast adipose tissue has long been a proposed strategy to decrease the risk of breast cancer development. Given the genomic instability associated with obesity, it is also possible that interventions focused on decreasing obesity-induced DNA damage in breast epithelial cells could prevent chromosomal defects, including mutations, that leads to tumor formation. Interventions that target body weight and pharmacological approaches that target obesity-induced changes in hormones and signaling pathways have shown promise in decreasing the risk of breast cancer **(** **Figure 4** **)**.

### Weight Loss Interventions

Weight loss, achieved through reduction of caloric intake, physical activity, or surgery, has been examined as a risk reduction strategy in both preclinical models and in human studies. The 2016 IARC working group’s review of body weight and cancer risk literature determined that weight loss, either through changes in lifestyle or by bariatric surgery, may reduce breast cancer risk, although the number and quality of the studies were deemed “insufficient for formal evaluation” (15). The mixed outcomes of weight loss studies can be explained in part by the wide variety of methods used to achieve weight loss, variability in degree of weight reduction, whether reduction in weight was sustained, and characteristics of study participants. Since the 2016 IARC report, a large-scale study analyzing data from more than 180,000 women across 10 prospective studies found that sustained weight loss in women over 50 was significantly associated with lower breast cancer risk compared with women who did not lose weight (128). Similar findings have been made in other studies where weight loss after menopause (129) or weight loss due to bariatric surgery (130) were associated with reduced breast cancer risk. Considerable observational data shows that exercise may also reduce breast cancer risk (131–134).

Mechanistically, beyond simply reducing the volume of dysfunctional adipose tissue, weight loss also mitigates many of the obesity-induced changes in adipose tissue that create a pro-carcinogenic microenvironment. In pre-clinical studies, weight loss after diet-induced obesity reduced mammary adipocyte size in association with decreases in adipose inflammation, aromatase expression and oxidative stress-related genes (135–137). Similarly, a number of human studies examining the impact of weight loss on inflammation have found reductions in inflammatory biomarkers including CRP, TNF, and IL-6 (138–140). In one study, weight loss induced by consumption of very low calorie diet significantly decreased inflammatory gene expression in subcutaneous adipose tissue while increasing anti-inflammatory gene expression (141). In a randomized controlled trial, Magkos et al. recently highlighted the dynamic nature of weight loss, showing that 5% weight loss was sufficient to illicit many metabolically beneficial effects, including downregulation of adipose tissue oxidative stress, yet was not sufficient to reduce adipose inflammation. Greater weight loss (16%), however, was associated with decreased adipose tissue inflammation and additional downregulation of transcriptional changes associated with oxidative stress (142). Furthermore, weight reduction has been associated with decreased levels of estradiol, leptin, and insulin along with an increase in adiponectin and steroid hormone binding globulin (SHBG), which binds free estradiol to reduce its availability in target tissues (138, 143, 144).

### Pharmacological Interventions

While lifestyle interventions that reduce weight have been reported to be effective at ameliorating the adipose tissue dysfunction associated with obesity and reducing breast cancer risk, major limitations of lifestyle approaches include the difficulty in adherence to diet and/or exercise regimens and the lack of sustainability of long-term weight loss (145, 146). Alternative risk reduction strategies involve pharmacological approaches that target inflammatory and hormonal changes in the obese breast microenvironment. For example, in a study of 5,078 women in the Nashville Breast Health Study (NBHS), regular use of nonsteroidal anti-inflammatory drugs (NSAIDs) was inversely associated with breast cancer risk in overweight women (147). Preclinical studies have shown NSAIDs to be effective at reducing or reversing obesity-induced adipose inflammation as well as decreasing adipose tissue leptin levels (148, 149). Leptin has also been targeted directly by Catalano et al. who reported on a novel leptin antagonist peptide which was found to inhibit breast cancer growth *in vitro* and *in vivo* (150).

Additionally, several drugs already used in the treatment of breast cancer inhibit estrogen signaling. Tamoxifen, a selective estrogen receptor modulator (SERM), competitively binds the estrogen receptor causing a conformational change that prevents binding by estrogen in the breast (151). However, while acting as an estrogen antagonist in the breast, tamoxifen acts as an estrogen agonist in other tissues, such as the endometrium and bone (151). These agonistic effects limit the feasibility of broadly employing tamoxifen in the preventative setting in obese women to inhibit estrogen signaling due to the potential of endometrial hyperplasia and other sides effects that may outweigh the preventative benefit. Nevertheless, several randomized control trials have found that tamoxifen and raloxifene, another SERM with fewer side effects, significantly reduce the risk of invasive breast cancer development in women who are at high risk (152–154). This led to the FDA approving both drugs for use in the preventative setting, making tamoxifen and raloxifene the only two breast cancer drugs approved for chemoprevention (155). Whether the elevated risk for breast cancer development associated with obesity in postmenopausal women represents a substantial enough risk to warrant the prophylactic use of these SERMs in this population remains to be studied. Other drugs that inhibit estrogen action include fulvestrant, an estrogen receptor degrader, which is a second line therapy approved for advanced ER+ breast cancer, and aromatase inhibitors (AIs). AIs are a commonly prescribed first line treatment of postmenopausal ER+ breast cancer, used with the goal of lowering levels of estrogens by preventing their synthesis from androgen precursors. Although it has been reported that AIs may lower the risk of breast cancer development in high-risk women, they are currently not approved for use in the prevention setting, due in part to significant side effects like osteoporosis and arthralgia, although additional prevention trials with AIs are ongoing (155, 156).

A more tenable strategy to reduce estrogen in the obese breast adipose microenvironment may be through use of metformin, a low cost, well-tolerated antidiabetic drug that has emerged as an intriguing drug candidate for breast cancer prevention. Although primarily used to reduce gluconeogenesis and increase insulin sensitivity in patients with type 2 diabetes, some, but not all, observational studies have reported lower incidence of breast cancer among diabetic patients taking metformin (157–159). While improving insulin sensitivity is likely to contribute to the decreased breast cancer risk, another important function of metformin in the context of obesity-associated breast cancer is its ability to inhibit aromatase expression in breast ASCs (160, 161). A recent preclinical study utilizing a model of postmenopausal carcinogen-induced breast cancer in obese rats found that metformin inhibited ASC aromatase expression in association with a reduction in size of existing mammary tumors and prevention of new tumor formation (162). Other studies have also supported an anti-tumor function of metformin, showing that treatment of several breast cancer cell lines inhibits growth, decreases colony formation, and induces cell cycle arrest (163–165). These actions are dependent on metformin-induced activation of AMP-activated protein kinase (AMPK), an important regulator of energy homeostasis (160, 164). Metformin may be especially beneficial in obese patients where AMPK activation also improves various aspects of obesity-associated adipose dysfunction, including decreasing adipose inflammation (166).

### Targeting Obesity-Induced DNA Damage

As reviewed above, many of the factors found to be reduced after weight loss or by pharmacological intervention, including estrogen, inflammatory mediators, and leptin have roles in promoting DNA damage and inhibiting repair processes. Therefore, it is possible that reduced DNA damage and improved DNA repair capacity represents an additional mechanistic link between the therapeutics that reduces these factors and lower risk of breast cancer. In a randomized controlled trial where participants were placed on a 6-month calorie restriction regimen, a significant decrease in DNA damage measured in blood by the comet assay was reported (167). Similar reductions in DNA damage have also been reported in blood and in lymphocytes of morbidly obese patients after weight loss surgery (168–170). Setayesh et al. showed in a mouse model of diet-induced obesity that weight loss significantly decreased DNA damage in the colon, liver, and testes in association with reductions in circulating levels of IL-6, MCP-1, leptin, and TNF and increased adiponectin (171). Interestingly, in a study of 220 healthy volunteers engaging in varying degrees of physical activity, physical activity at any intensity was associated with increased leukocyte repair capacity (172). To date, no studies have looked at the effects of weight loss on DNA damage or DNA repair capacity in normal breast epithelial cells. However, it stands to reason that reducing factors that have known genotoxic effects in the obese breast adipose microenvironment may be an effective strategy to prevent or reduce DNA damage in pre-neoplastic breast epithelial cells and consequently, mitigate the risk of breast tumorigenesis.

## Discussion

Abundant evidence demonstrates the importance of the crosstalk between obese breast adipose tissue and neighboring normal and neoplastic breast epithelial cells. Excess caloric intake and subsequent weight gain initiates a cascade of effects in breast adipose tissue beginning with adipocyte hypertrophy and progressing to oxidative stress, chronic inflammation, altered adipokine secretion, and elevated estrogen production by ASCs. While these are major, well-established changes in the breast microenvironment, a wide variety of additional changes occur in the setting of obesity, which can also promote breast cancer and have been reviewed by others, for example extracellular matrix stiffening, immune cell dysfunction, and dysregulation of insulin. The current review highlights the effects of breast adipose-derived estrogen, inflammatory mediators, leptin and decreased adiponectin on two aspects of breast cancer: initial events leading to carcinogenesis and progression. These factors have the ability to initiate breast cancer through regulation of oxidative stress, DNA damage, and DNA repair response. Further large-scale studies are warranted to elucidate whether there is a direct mechanistic link between adipose derived factors and breast tumorigenesis through stimulation of genomic instability. Promotion of breast cancer growth occurs through regulation of pro-proliferative and anti-apoptotic transcriptional programs in cancer cells and through effects on microenvironment oxidative stress. Targeting obese breast adipose dysfunction with weight reduction or pharmacological approaches has so far shown promise at decreasing breast cancer risk. With obesity rates expected to reach over 50% in the next 10 years in the U.S., additional studies aimed at disrupting the crosstalk between obese breast adipose tissue and neighboring breast epithelial cells may elucidate novel, effective prevention strategies for obese women at elevated risk for breast cancer development.

## Author Contributions

PB prepared first draft, KB reviewed and edited. All authors contributed to the article and approved the submitted version. 

## Funding

This work was supported by NIH R01 CA215797, Anne Moore Breast Cancer Research Fund, and NIH 5 F31 CA236306-02.

## Conflict of Interest

The authors declare that the research was conducted in the absence of any commercial or financial relationships that could be construed as a potential conflict of interest.
